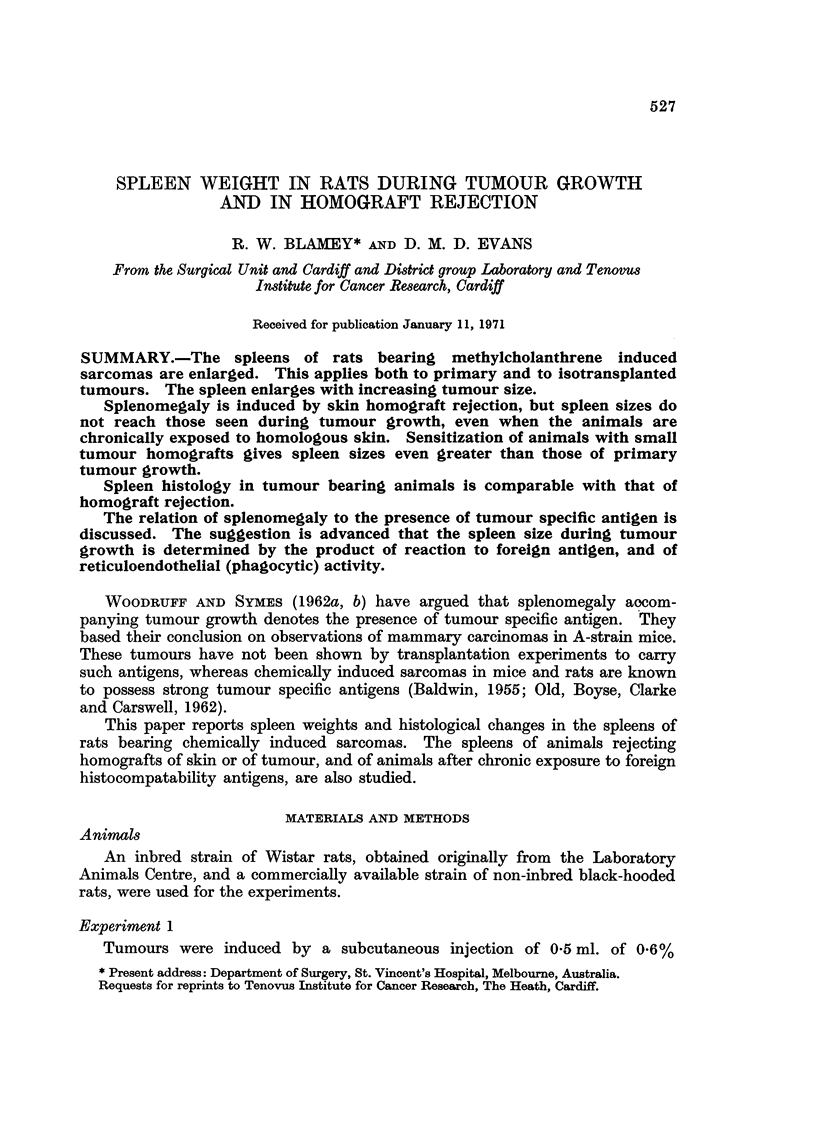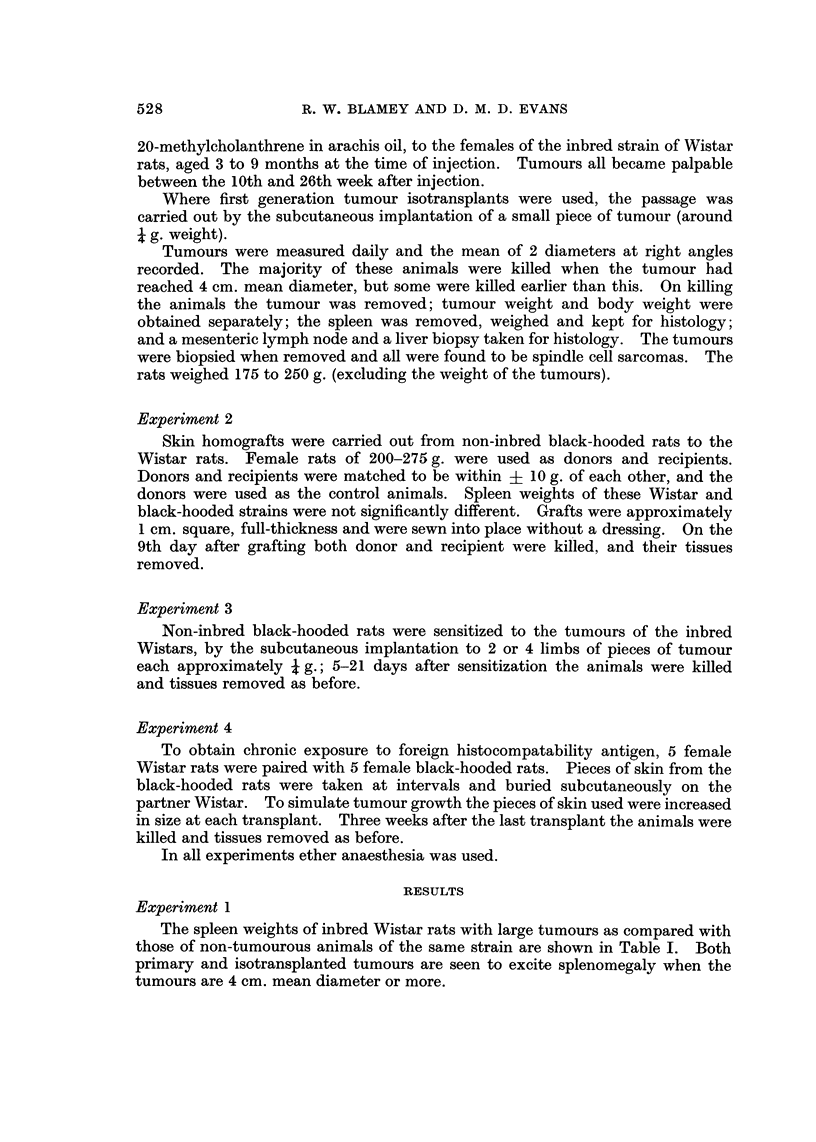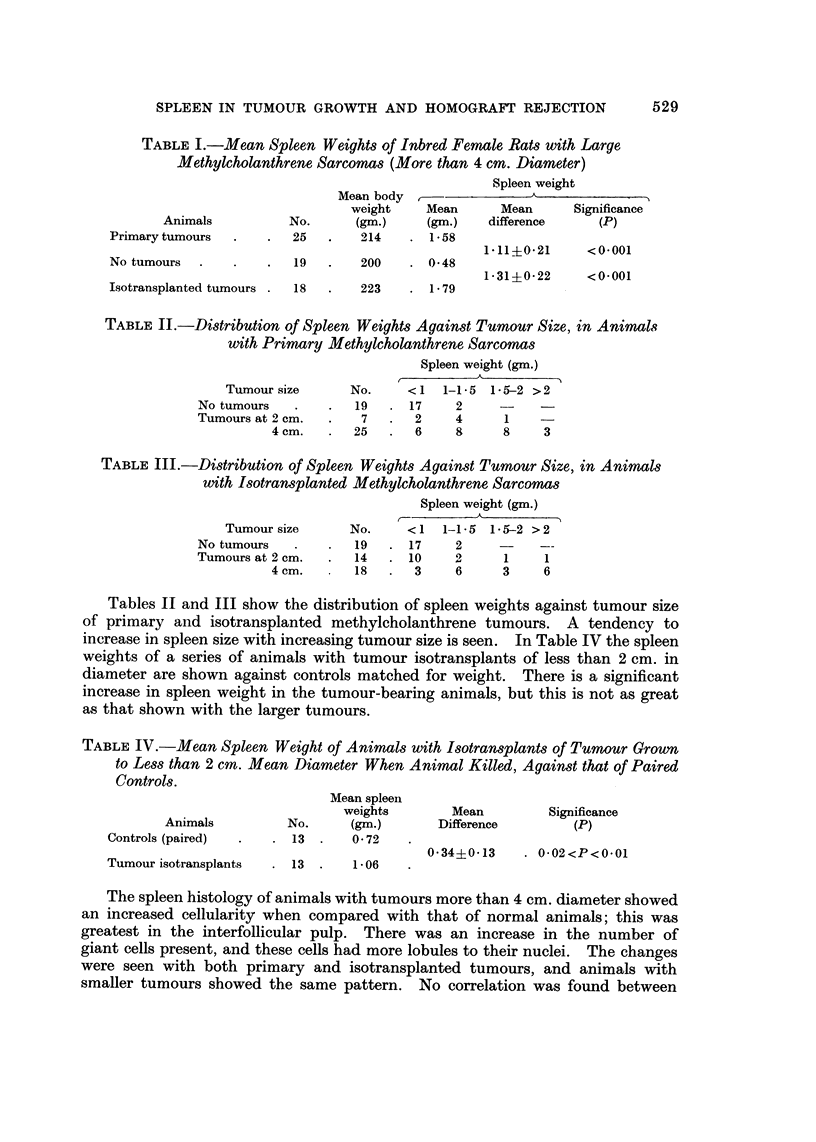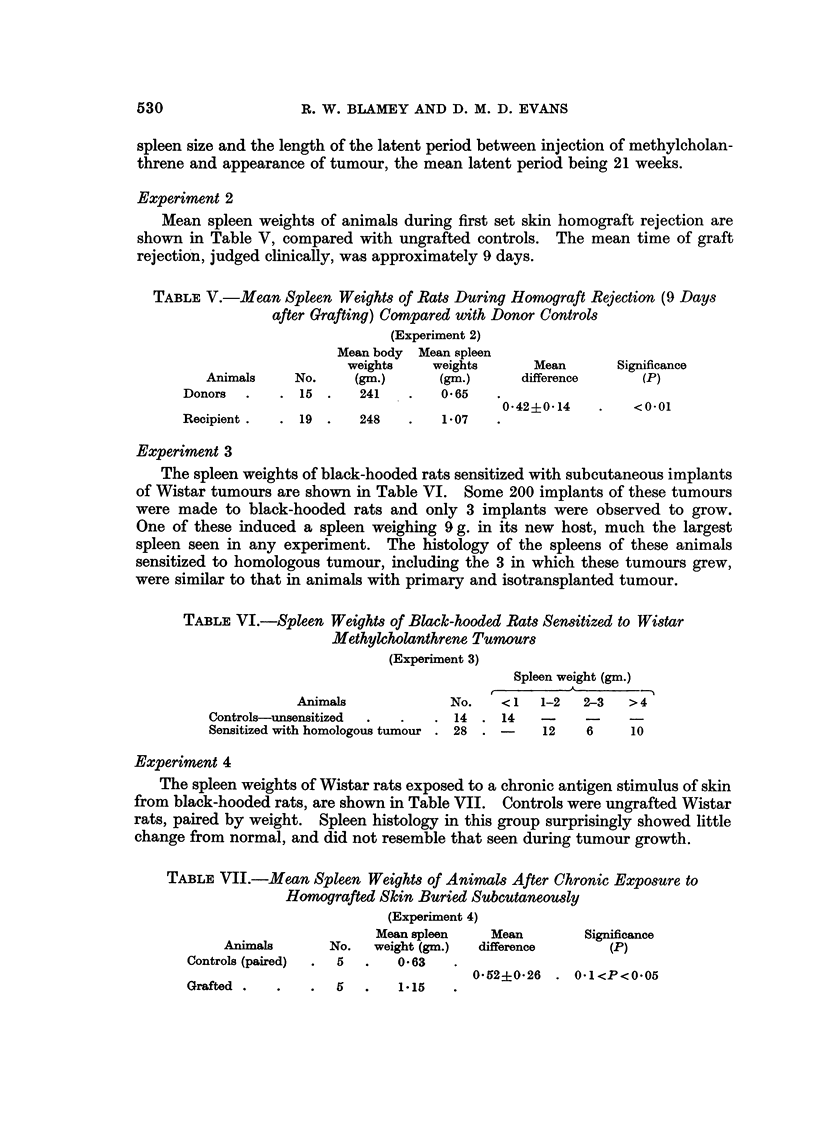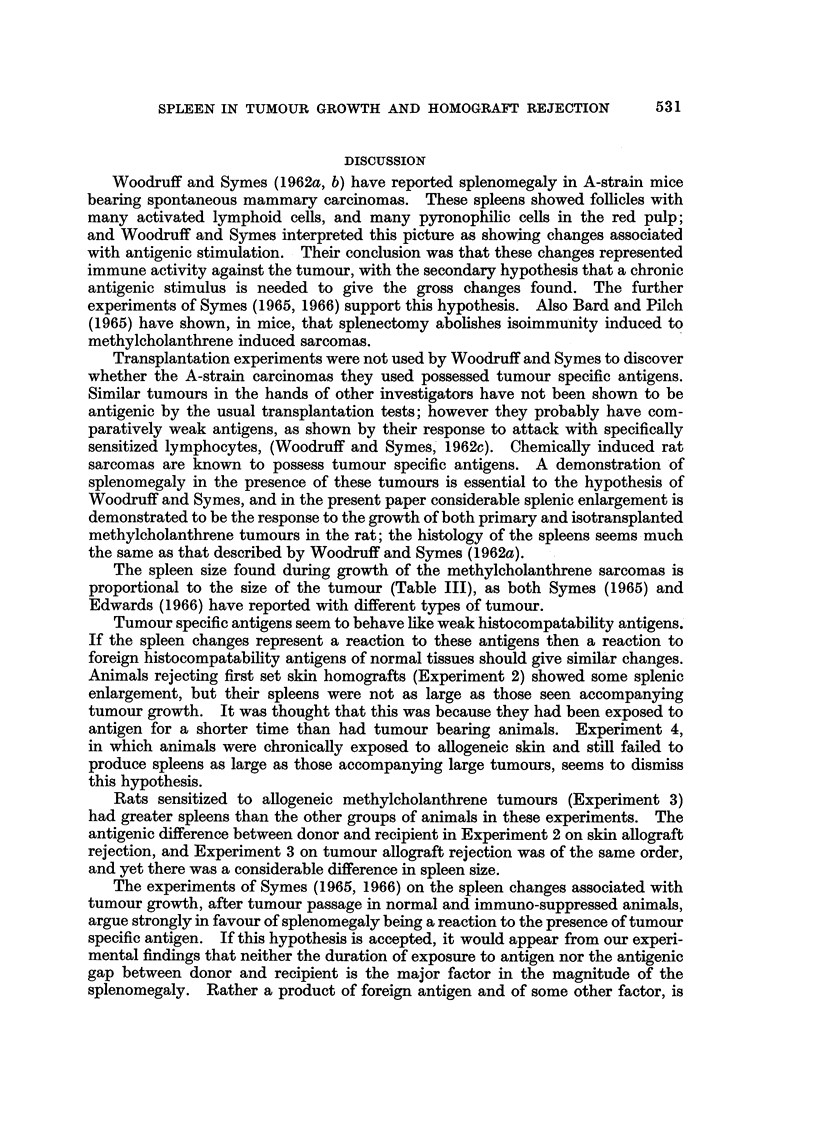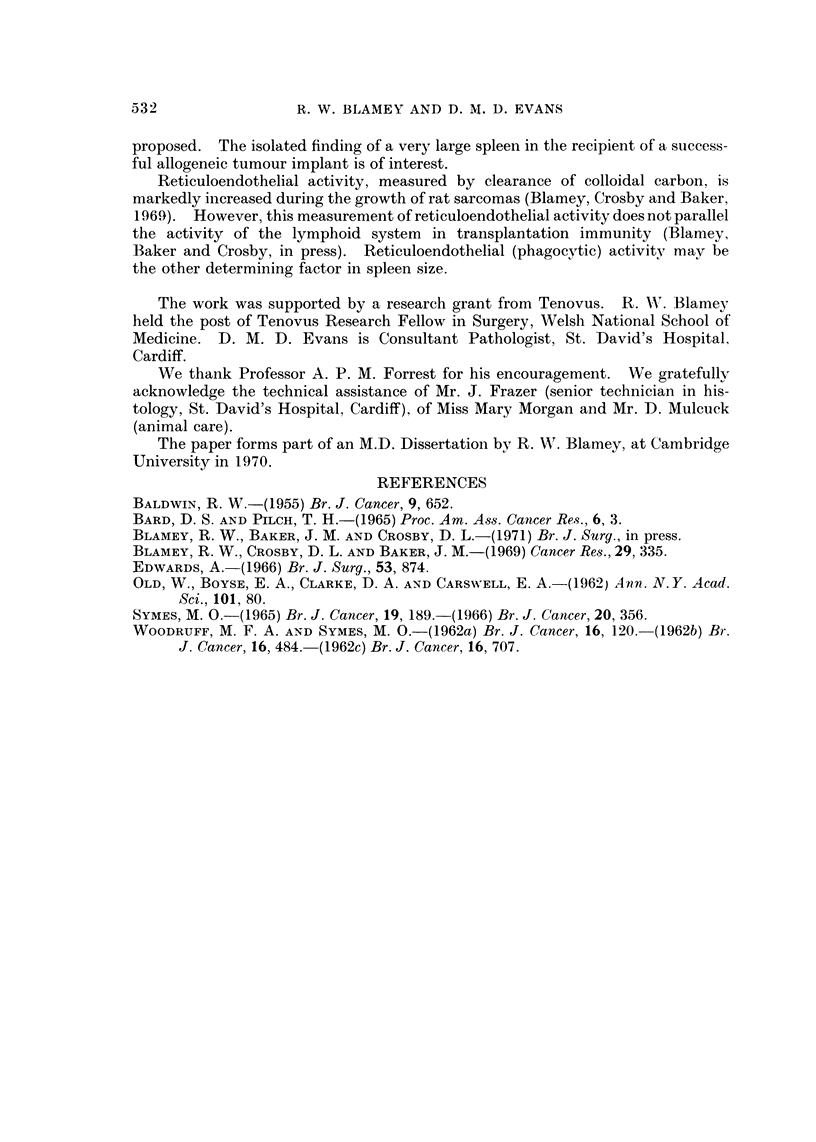# Spleen Weight in Rats During Tumour Growth and in Homograft Rejection

**DOI:** 10.1038/bjc.1971.67

**Published:** 1971-09

**Authors:** R. W. Blamey, D. M. D. Evans

## Abstract

The spleens of rats bearing methylcholanthrene induced sarcomas are enlarged. This applies both to primary and to isotransplanted tumours. The spleen enlarges with increasing tumour size.

Splenomegaly is induced by skin homograft rejection, but spleen sizes do not reach those seen during tumour growth, even when the animals are chronically exposed to homologous skin. Sensitization of animals with small tumour homografts gives spleen sizes even greater than those of primary tumour growth.

Spleen histology in tumour bearing animals is comparable with that of homograft rejection.

The relation of splenomegaly to the presence of tumour specific antigen is discussed. The suggestion is advanced that the spleen size during tumour growth is determined by the product of reaction to foreign antigen, and of reticuloendothelial (phagocytic) activity.


### SPLEEN WEIGHT IN RATS DURING TUMOUR GROWTH AND IN HOMOGRAFT REJECTION

R. W. BLAMEY* AND D. M. D. EVANS

*From the Surgical Unit and Cardiff and District group Laboratory and Tenovus Institute for Cancer Research, Cardiff*

Received for publication January 11, 1971

**SUMMARY.—The spleens of rats bearing methylcholanthrene induced sarcomas are enlarged. This applies both to primary and to isotransplanted tumours. The spleen enlarges with increasing tumour size.**

**Splenomegaly is induced by skin homograft rejection, but spleen sizes do not reach those seen during tumour growth, even when the animals are chronically exposed to homologous skin. Sensitization of animals with small tumour homografts gives spleen sizes even greater than those of primary tumour growth.**

**Spleen histology in tumour bearing animals is comparable with that of homograft rejection.**

**The relation of splenomegaly to the presence of tumour specific antigen is discussed. The suggestion is advanced that the spleen size during tumour growth is determined by the product of reaction to foreign antigen, and of reticuloendothelial (phagocytic) activity.**

WOODRUFF AND SYMES (1962*a*, *b*) have argued that splenomegaly accompanying tumour growth denotes the presence of tumour specific antigen. They based their conclusion on observations of mammary carcinomas in A-strain mice. These tumours have not been shown by transplantation experiments to carry such antigens, whereas chemically induced sarcomas in mice and rats are known to possess strong tumour specific antigens (Baldwin, 1955; Old, Boyse, Clarke and Carswell, 1962).

This paper reports spleen weights and histological changes in the spleens of rats bearing chemically induced sarcomas. The spleens of animals rejecting homografts of skin or of tumour, and of animals after chronic exposure to foreign histocompatability antigens, are also studied.

### MATERIALS AND METHODS

### *Animals*

An inbred strain of Wistar rats, obtained originally from the Laboratory Animals Centre, and a commercially available strain of non-inbred black-hooded rats, were used for the experiments.

### *Experiment* 1

Tumours were induced by a subcutaneous injection of 0·5 ml. of 0·6%

* Present address: Department of Surgery, St. Vincent's Hospital, Melbourne, Australia.
Requests for reprints to Tenovus Institute for Cancer Research, The Heath, Cardiff.

20-methylcholanthrene in arachis oil, to the females of the inbred strain of Wistar rats, aged 3 to 9 months at the time of injection. Tumours all became palpable between the 10th and 26th week after injection.

Where first generation tumour isotransplants were used, the passage was carried out by the subcutaneous implantation of a small piece of tumour (around $\frac{1}{4}$ g. weight).

Tumours were measured daily and the mean of 2 diameters at right angles recorded. The majority of these animals were killed when the tumour had reached 4 cm. mean diameter, but some were killed earlier than this. On killing the animals the tumour was removed; tumour weight and body weight were obtained separately; the spleen was removed, weighed and kept for histology; and a mesenteric lymph node and a liver biopsy taken for histology. The tumours were biopsied when removed and all were found to be spindle cell sarcomas. The rats weighed 175 to 250 g. (excluding the weight of the tumours).

*Experiment* 2

Skin homografts were carried out from non-inbred black-hooded rats to the Wistar rats. Female rats of 200–275 g. were used as donors and recipients. Donors and recipients were matched to be within $\pm$ 10 g. of each other, and the donors were used as the control animals. Spleen weights of these Wistar and black-hooded strains were not significantly different. Grafts were approximately 1 cm. square, full-thickness and were sewn into place without a dressing. On the 9th day after grafting both donor and recipient were killed, and their tissues removed.

*Experiment* 3

Non-inbred black-hooded rats were sensitized to the tumours of the inbred Wistars, by the subcutaneous implantation to 2 or 4 limbs of pieces of tumour each approximately $\frac{1}{4}$ g.; 5–21 days after sensitization the animals were killed and tissues removed as before.

*Experiment* 4

To obtain chronic exposure to foreign histocompatability antigen, 5 female Wistar rats were paired with 5 female black-hooded rats. Pieces of skin from the black-hooded rats were taken at intervals and buried subcutaneously on the partner Wistar. To simulate tumour growth the pieces of skin used were increased in size at each transplant. Three weeks after the last transplant the animals were killed and tissues removed as before.

In all experiments ether anaesthesia was used.

### RESULTS

*Experiment* 1

The spleen weights of inbred Wistar rats with large tumours as compared with those of non-tumourous animals of the same strain are shown in Table I. Both primary and isotransplanted tumours are seen to excite splenomegaly when the tumours are 4 cm. mean diameter or more.

TABLE I.—*Mean Spleen Weights of Inbred Female Rats with Large Methylcholanthrene Sarcomas (More than 4 cm. Diameter)*

| Animals | No. | Mean body weight (gm.) | Spleen weight: Mean (gm.) | Mean difference | Significance (*P*) |
|---|---|---|---|---|---|
| Primary tumours | 25 | 214 | 1·58 | | |
| | | | | 1·11±0·21 | <0·001 |
| No tumours | 19 | 200 | 0·48 | | |
| | | | | 1·31±0·22 | <0·001 |
| Isotransplanted tumours | 18 | 223 | 1·79 | | |

TABLE II.—*Distribution of Spleen Weights Against Tumour Size, in Animals with Primary Methylcholanthrene Sarcomas*

| Tumour size | No. | Spleen weight (gm.): <1 | 1–1·5 | 1·5–2 | >2 |
|---|---|---|---|---|---|
| No tumours | 19 | 17 | 2 | — | — |
| Tumours at 2 cm. | 7 | 2 | 4 | 1 | — |
| 4 cm. | 25 | 6 | 8 | 8 | 3 |

TABLE III.—*Distribution of Spleen Weights Against Tumour Size, in Animals with Isotransplanted Methylcholanthrene Sarcomas*

| Tumour size | No. | Spleen weight (gm.): <1 | 1–1·5 | 1·5–2 | >2 |
|---|---|---|---|---|---|
| No tumours | 19 | 17 | 2 | — | — |
| Tumours at 2 cm. | 14 | 10 | 2 | 1 | 1 |
| 4 cm. | 18 | 3 | 6 | 3 | 6 |

Tables II and III show the distribution of spleen weights against tumour size of primary and isotransplanted methylcholanthrene tumours. A tendency to increase in spleen size with increasing tumour size is seen. In Table IV the spleen weights of a series of animals with tumour isotransplants of less than 2 cm. in diameter are shown against controls matched for weight. There is a significant increase in spleen weight in the tumour-bearing animals, but this is not as great as that shown with the larger tumours.

TABLE IV.—*Mean Spleen Weight of Animals with Isotransplants of Tumour Grown to Less than 2 cm. Mean Diameter When Animal Killed, Against that of Paired Controls.*

| Animals | No. | Mean spleen weights (gm.) | Mean Difference | Significance (*P*) |
|---|---|---|---|---|
| Controls (paired) | 13 | 0·72 | | |
| | | | 0·34±0·13 | $0·02 < P < 0·01$ |
| Tumour isotransplants | 13 | 1·06 | | |

The spleen histology of animals with tumours more than 4 cm. diameter showed an increased cellularity when compared with that of normal animals; this was greatest in the interfollicular pulp. There was an increase in the number of giant cells present, and these cells had more lobules to their nuclei. The changes were seen with both primary and isotransplanted tumours, and animals with smaller tumours showed the same pattern. No correlation was found between spleen size and the length of the latent period between injection of methylcholanthrene and appearance of tumour, the mean latent period being 21 weeks.

*Experiment* 2

Mean spleen weights of animals during first set skin homograft rejection are shown in Table V, compared with ungrafted controls. The mean time of graft rejection, judged clinically, was approximately 9 days.

TABLE V.—*Mean Spleen Weights of Rats During Homograft Rejection* (9 *Days after Grafting*) *Compared with Donor Controls*

(Experiment 2)

| Animals | No. | Mean body weights (gm.) | Mean spleen weights (gm.) | Mean difference | Significance (*P*) |
|---|---|---|---|---|---|
| Donors | 15 | 241 | 0·65 | 0·42±0·14 | <0·01 |
| Recipient | 19 | 248 | 1·07 | | |

*Experiment* 3

The spleen weights of black-hooded rats sensitized with subcutaneous implants of Wistar tumours are shown in Table VI. Some 200 implants of these tumours were made to black-hooded rats and only 3 implants were observed to grow. One of these induced a spleen weighing 9 g. in its new host, much the largest spleen seen in any experiment. The histology of the spleens of these animals sensitized to homologous tumour, including the 3 in which these tumours grew, were similar to that in animals with primary and isotransplanted tumour.

TABLE VI.—*Spleen Weights of Black-hooded Rats Sensitized to Wistar Methylcholanthrene Tumours*

(Experiment 3)

| Animals | No. | Spleen weight (gm.) | | | |
|---|---|---|---|---|---|
| | | <1 | 1–2 | 2–3 | >4 |
| Controls—unsensitized | 14 | 14 | — | — | — |
| Sensitized with homologous tumour | 28 | — | 12 | 6 | 10 |

*Experiment* 4

The spleen weights of Wistar rats exposed to a chronic antigen stimulus of skin from black-hooded rats, are shown in Table VII. Controls were ungrafted Wistar rats, paired by weight. Spleen histology in this group surprisingly showed little change from normal, and did not resemble that seen during tumour growth.

TABLE VII.—*Mean Spleen Weights of Animals After Chronic Exposure to Homografted Skin Buried Subcutaneously*

(Experiment 4)

| Animals | No. | Mean spleen weight (gm.) | Mean difference | Significance (*P*) |
|---|---|---|---|---|
| Controls (paired) | 5 | 0·63 | 0·52±0·26 | 0·1<*P*<0·05 |
| Grafted | 5 | 1·15 | | |

### DISCUSSION

Woodruff and Symes (1962*a*, *b*) have reported splenomegaly in A-strain mice bearing spontaneous mammary carcinomas. These spleens showed follicles with many activated lymphoid cells, and many pyronophilic cells in the red pulp; and Woodruff and Symes interpreted this picture as showing changes associated with antigenic stimulation. Their conclusion was that these changes represented immune activity against the tumour, with the secondary hypothesis that a chronic antigenic stimulus is needed to give the gross changes found. The further experiments of Symes (1965, 1966) support this hypothesis. Also Bard and Pilch (1965) have shown, in mice, that splenectomy abolishes isoimmunity induced to methylcholanthrene induced sarcomas.

Transplantation experiments were not used by Woodruff and Symes to discover whether the A-strain carcinomas they used possessed tumour specific antigens. Similar tumours in the hands of other investigators have not been shown to be antigenic by the usual transplantation tests; however they probably have comparatively weak antigens, as shown by their response to attack with specifically sensitized lymphocytes, (Woodruff and Symes, 1962*c*). Chemically induced rat sarcomas are known to possess tumour specific antigens. A demonstration of splenomegaly in the presence of these tumours is essential to the hypothesis of Woodruff and Symes, and in the present paper considerable splenic enlargement is demonstrated to be the response to the growth of both primary and isotransplanted methylcholanthrene tumours in the rat; the histology of the spleens seems much the same as that described by Woodruff and Symes (1962*a*).

The spleen size found during growth of the methylcholanthrene sarcomas is proportional to the size of the tumour (Table III), as both Symes (1965) and Edwards (1966) have reported with different types of tumour.

Tumour specific antigens seem to behave like weak histocompatability antigens. If the spleen changes represent a reaction to these antigens then a reaction to foreign histocompatability antigens of normal tissues should give similar changes. Animals rejecting first set skin homografts (Experiment 2) showed some splenic enlargement, but their spleens were not as large as those seen accompanying tumour growth. It was thought that this was because they had been exposed to antigen for a shorter time than had tumour bearing animals. Experiment 4, in which animals were chronically exposed to allogeneic skin and still failed to produce spleens as large as those accompanying large tumours, seems to dismiss this hypothesis.

Rats sensitized to allogeneic methylcholanthrene tumours (Experiment 3) had greater spleens than the other groups of animals in these experiments. The antigenic difference between donor and recipient in Experiment 2 on skin allograft rejection, and Experiment 3 on tumour allograft rejection was of the same order, and yet there was a considerable difference in spleen size.

The experiments of Symes (1965, 1966) on the spleen changes associated with tumour growth, after tumour passage in normal and immuno-suppressed animals, argue strongly in favour of splenomegaly being a reaction to the presence of tumour specific antigen. If this hypothesis is accepted, it would appear from our experimental findings that neither the duration of exposure to antigen nor the antigenic gap between donor and recipient is the major factor in the magnitude of the splenomegaly. Rather a product of foreign antigen and of some other factor, is proposed. The isolated finding of a very large spleen in the recipient of a successful allogeneic tumour implant is of interest.

Reticuloendothelial activity, measured by clearance of colloidal carbon, is markedly increased during the growth of rat sarcomas (Blamey, Crosby and Baker, 1969). However, this measurement of reticuloendothelial activity does not parallel the activity of the lymphoid system in transplantation immunity (Blamey, Baker and Crosby, in press). Reticuloendothelial (phagocytic) activity may be the other determining factor in spleen size.

The work was supported by a research grant from Tenovus. R. W. Blamey held the post of Tenovus Research Fellow in Surgery, Welsh National School of Medicine. D. M. D. Evans is Consultant Pathologist, St. David's Hospital, Cardiff.

We thank Professor A. P. M. Forrest for his encouragement. We gratefully acknowledge the technical assistance of Mr. J. Frazer (senior technician in histology, St. David's Hospital, Cardiff), of Miss Mary Morgan and Mr. D. Mulcuck (animal care).

The paper forms part of an M.D. Dissertation by R. W. Blamey, at Cambridge University in 1970.

### REFERENCES

Baldwin, R. W.—(1955) *Br. J. Cancer*, **9**, 652.

Bard, D. S. and Pilch, T. H.—(1965) *Proc. Am. Ass. Cancer Res.*, **6**, 3.

Blamey, R. W., Baker, J. M. and Crosby, D. L.—(1971) *Br. J. Surg.*, in press.

Blamey, R. W., Crosby, D. L. and Baker, J. M.—(1969) *Cancer Res.*, **29**, 335.

Edwards, A.—(1966) *Br. J. Surg.*, **53**, 874.

Old, W., Boyse, E. A., Clarke, D. A. and Carswell, E. A.—(1962) *Ann. N.Y. Acad. Sci.*, **101**, 80.

Symes, M. O.—(1965) *Br. J. Cancer*, **19**, 189.—(1966) *Br. J. Cancer*, **20**, 356.

Woodruff, M. F. A. and Symes, M. O.—(1962*a*) *Br. J. Cancer*, **16**, 120.—(1962*b*) *Br. J. Cancer*, **16**, 484.—(1962*c*) *Br. J. Cancer*, **16**, 707.